# Swallowing transit times and valleculae residue in stable chronic obstructive pulmonary disease

**DOI:** 10.1186/1471-2466-14-62

**Published:** 2014-04-16

**Authors:** Rosane de Deus Chaves, Fernanda Chiarion Sassi, Laura Davison Mangilli, Shri Krishna Jayanthi, Alberto Cukier, Bruno Zilberstein, Claudia Regina Furquim de Andrade

**Affiliations:** 1 Department of Physiotherapy, Speech-language and Hearing Sciences, and Occupational Therapy, School of Medicine, University of São Paulo, Rua Ovídeo Pires de Campos, 186, Cerqueira César, CEP 054030-010 São Paulo, SP, Brazil; 2 Medical Investigation Laboratory (LIM 34) – Rehabilitation Sciences, School of Medicine, University of São Paulo, Rua Ovídeo Pires de Campos, 186, Cerqueira César, CEP 054030-010 São Paulo, SP, Brazil; 3 Institute of Radiology, Hospital das Clínicas, School of Medicine, University of São Paulo, Rua Ovídeo Pires de Campos, 186, Cerqueira César, CEP 054030-010 São Paulo, SP, Brazil; 4 Pneumology Division, Heart Institute of Hospital das Clínicas, School of Medicine, University of São Paulo, Rua Ovídeo Pires de Campos, 186, Cerqueira César, CEP 054030-010 São Paulo, SP, Brazil; 5 Digestive Surgery Division, School of Medicine, University of São Paulo, Rua Ovídeo Pires de Campos, 186, Cerqueira César, CEP 054030-010 São Paulo, SP, Brazil

**Keywords:** COPD, Videofluoroscopy, Swallowing, Swallowing disorders

## Abstract

**Background:**

Breathing and swallowing are physiologically linked to ensure effortless gas exchange during oronasal breathing and to prevent aspiration during swallowing. Studies have indicated consistent aspiration in chronic obstructive pulmonary disease, mainly related to delayed swallowing reflex and problems with lingual propulsion and pharyngeal peristalsis as a result of bilateral weakness and incoordination of the related muscles. The purpose of the present study was to evaluate swallowing transit times and valleculae residue characteristics of stable COPD patients who have no swallowing complaints.

**Methods:**

Our study population included 20 stable patients with COPD and no swallowing complaints and 20 healthy controls. Swallowing was assessed through videofluoroscopic examination and involved the analysis of the following parameters: (1) pharyngeal stages of deglutition; (2) the duration of bolus movement through the oral cavity and pharynx (i.e. transit times); (3) valleculae residue ratio; (4) penetration/aspiration.

**Results:**

Participants of the study did not present any signs of penetration-aspiration for any of the tested consistencies. Patients with COPD presented longer pharyngeal transit times during the ingestion of the liquid consistency and during the ingestion of the paste consistency. Regarding the duration of tongue base contact with the posterior pharyngeal wall, COPD patients also presented longer durations for the liquid and paste consistencies. No significant difference was observed for the distribution of individuals among the different valleculae residue severity levels.

**Conclusions:**

Our study suggests that stable COPD patients may present physiological adaptations as a protective swallowing maneuver to avoid aspiration/penetration of pharyngeal contents. Moreover, valleculae residue cannot be seen as an isolated factor when trying to explain swallowing alterations in this population.

## Background

Chronic obstructive pulmonary disease (COPD) is a major cause of chronic morbidity and mortality and represents a substantial economic and social burden throughout the world [1]. It is the fourth-leading cause of death in the United States and Europe and the burden of COPD is likely to worsen as the population continues to age [2]. In Brazil, COPD is the third-leading cause of death, having presented a 12% increase between 2005 and 2010 [3]. This means that nearly 40,000 deaths/year are caused by COPD. It is estimated that 5 to 6 million Brazilians have COPD, and patients tend to be mostly concentrated in the age groups above 50 years [4].

Poor nutritional status of COPD patients has been related to adverse effects that may contribute to complications and increased mortality [5]. Malnutrition is due to decreased food intake, but mostly to a higher resting metabolic rate [6]. Dysphagia is usually a precipitating factor for malnutrition [7-10]. To date, little attention has been given to the investigation of swallowing disorders in patients with COPD. Prevalence of dysphagia in COPD varies considerably across studies [11,12] and the basis for dysphagia is still uncertain.

One of the reasons for patients with COPD to be considered at risk for dysphagia, or at least for presenting some swallowing impairment is the fact that swallowing and respiration are coordinated functions, i.e. respiration halts when the swallowing reflex is triggered [13]. Breathing and swallowing are physiologically linked to ensure effortless gas exchange during oronasal breathing and to prevent aspiration during swallowing [14]. The literature suggests that the respiratory system may have a regulatory function related to swallowing and that subglottic air pressure may be important for swallowing integrity [15]. According to Gross et al. [15], feedback mechanisms that use afferent information from the subglottis may be more readily accessed at higher lung volumes or during exhalation when it is easiest to produce positive air pressure.

Coelho [16] suggested that COPD may weaken the strength to swallow. Muscle structure and function are frequently abnormal in patients COPD [17,18]. Muscle dysfunction is defined as the loss of at least one of the two main muscle properties: strength and endurance [19]. Studies have indicated consistent aspiration in COPD patients, mainly related to delayed swallowing reflex [16,20] and problems with lingual and pharyngeal peristalsis as a result of bilateral weakness and incoordination of the related muscles [16]. When combined with an impaired ability to use expired air to clear the larynx and protect the airway, a weak swallow may contribute to an increased risk for aspiration of pharyngeal contents, and may consequently lead to aspiration pneumonia [21]. More recently, Cvejic et al. [22] provided important evidence of aspiration during swallowing in patients with stable moderate COPD assessed by videofluoroscopy while respiration was monitored. They found that patients with COPD were more likely to have either penetration of pharyngeal contents into the larynx or actual aspiration when swallowing large volumes of liquid consistency (i.e. 100 ml).

Moreover, impaired laryngopharyngeal sensitivity may be clinically important in COPD patients, especially for those who have high rates of hospitalization related to lower respiratory tract infection [21]. Although the cause of impaired laryngopharyngeal sensitivity remains unclear, a few hypothesis have been risen: the use of inhaled coriticosteroids and anticholinergics may have an effect on the sensory mucosa of the laryngopharynx; laryngeal edema caused by smoking and presence of chronic cough, commonly reported in COPD patients, may contribute to reduced sensation [21].

The purpose of the present study was to evaluate swallowing transit times and valleculae residue characteristics of stable COPD patients who have no swallowing complaints. Considering the alterations described above, our hypothesis was that although stable and not presenting swallowing complaints, patients with COPD should present signs of altered swallowing (i.e. larger amounts of valleculae residue and alterations in swallowing transit times) when compared to their healthy pairs.

## Methods

### Study participants

An observational, descriptive, cross-sectional study was conducted with COPD patients who were seen at the COPD Ambulatory of a large Brazilian School Hospital (*Hospital das Clínicas*), and healthy volunteers. The study design was approved by the Ethics Committee for the Analysis of Research Projects (CAPPesq HCFMUSP no. 0074/08). Prior to their enrollment, all participants were informed of the purpose and procedures, after which all gave written informed consent.

The assessment of swallowing was only performed in the subjects with COPD after verifying if they were in a stable condition, i.e. no exacerbation of symptoms for at least the past 30 days, as confirmed by the medical team [23,24]. The inclusion criteria for patients with COPD involved the diagnosis of COPD according to the Global Initiative for Chronic Obstructive Lung Disease [25], ages between 50 and 65 years, absence of self-report of swallowing/feeding difficulties and absence of prior swallowing management. The exclusion criteria for subjects with COPD were as follows: comorbidities and/or past history of disease affecting the clinical course of COPD and swallowing mechanisms (e.g. additional respiratory diseases, malignant diseases, otorhinolaryngeal diseases, stroke, or active inflammatory disorders other than COPD); withdrawal of COPD-related medication; use of oxygen and/or ventilation during the research period.

The healthy control subjects were matched for age and gender to the COPD patients and were recruited from individuals who visited the Hospital for health status check-ups. All controls were non-smokers and did not present previous smoking habits. The exclusion criteria for the control subjects were as follows: presence of comorbidities of respiratory disorders including COPD; presence of comorbidities and/or a past history of disease affecting the clinical course of swallowing mechanisms (e.g. malignant diseases, otorhinolaryngeal diseases, stroke, or active inflammatory disease); self-report of swallowing/feeding difficulties and prior swallowing management.

**Figure 1:**
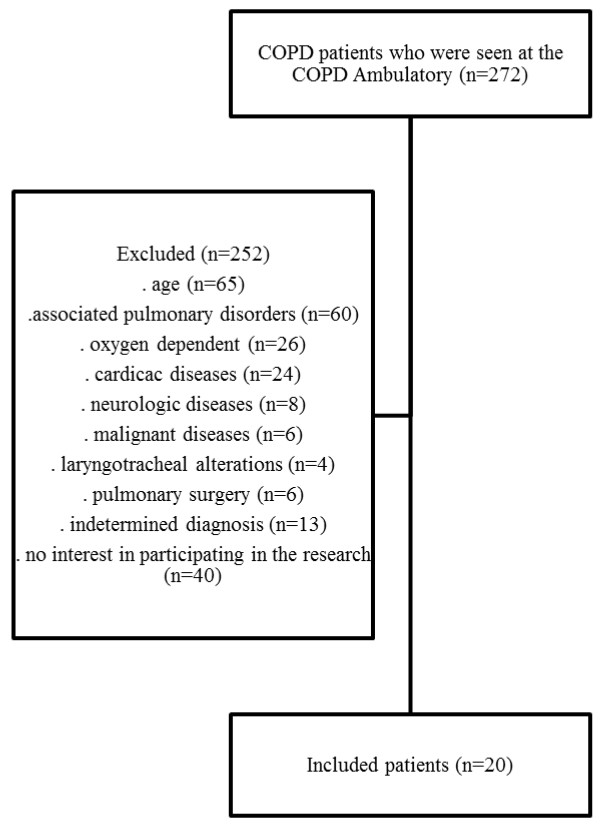
Study population with COPD.

Thus, our final study population included 20 patients with COPD, 10 males and 10 females, with a mean age of 59 years (±4.1), and 20 healthy controls, 10 males and 10 females, also with a mean age of 59 years (±4.4) (Figure 1). COPD patients presented the following smoking habits: males 57.8(±40.0) packs per year; females 50.8(±24.9) packs per year.

### Measures

The variables considered to be baseline characteristics were spirometry (i.e. forced expiratory volume during the first second - FEV_1_) and the body mass index (BMI) obtained according to the National Institutes of Health and the National Heart, Lung and Blood Institute (kg/m^2^).

Swallowing was assessed through videofluoroscopic examination (VFSS). The fluoroscopy unit used for this study was the GE Medical Systems, ADVANTX. All VFSS were performed in a lateral plane by a videofluoroscopy-trained radiographer and two speech and language therapists at the same Hospital. Participants remained seated, at an angle of 90°, with their heads positioned horizontally according to the Frankfort plane (i.e. head parallel to ground, considering the position of the inferior margin of the left orbit and the upper margin of each external auditory meatus), during the entire exam. Liquid barium (Opti-bar) with 100% w/v was used. The protocol adopted for the swallowing assessment involved the ingestion of different food consistencies and was determined by the Radiology Service. This assessment protocol is used routinely at our Hospital for investigating swallowing characteristics, especially the presence of aspiration, and includes de following:

a) swallow of 3, 5 and 10 ml of liquid consistency (50% water and 50% barium). The mixture was graded on a disposable syringe and offered to the subjects in a cup;

b) swallow of 7 ml of paste consistency (20 ml of an homogenous paste - apple puree by Néstle - and 5 ml of barium). The mixture volume was measured using a graded disposable syringe and was offered to the subjects on a spoon;

c) swallow of solid consistency (half “salt and water” biscuit dipped in 2.5 ml of the prepared paste consistency).

For each one of the consistencies, the participants were asked to swallow in their habitual manner in order to avoid possible interferences in their swallowing process. VFSS images were recorded on a VHS recorder and later digitalized in DVDs at 30 frames per second. In order to digitalize the obtained images, the software TOO DVD Ripper Platinum v4.0.64 was used. Digitalized images were then viewed using the software VitualDub v1.8.6, allowing a slow motion frame by frame analysis and enabling the definition of the following: (1) pharyngeal stages of deglutition; (2) the duration of bolus movement through and pharynx (i.e. transit times); (3) valleculae residue ratio [26]; (4) penetration/aspiration.

### VFSS analysis

Swallowing was analyzed by reviewing the digitalized images of each swallow in slow motion. Temporal measures of the critical physiologic events in the oropharyngeal swallow and measurement of valeculae residue for each swallow was performed and involved the following:

$$\left(\mathrm{area}\;\mathrm{of}\;\mathrm{residue}/\mathrm{area}\;\mathrm{of}\;\mathrm{valleculae}\right)\;\times \;100\%=\mathrm{percentage}\;\mathrm{of}\;\mathrm{the}\;\mathrm{valleculae}\;\mathrm{occupied}\;\mathrm{by}\;\mathrm{residue}$$

**Figure 2:**
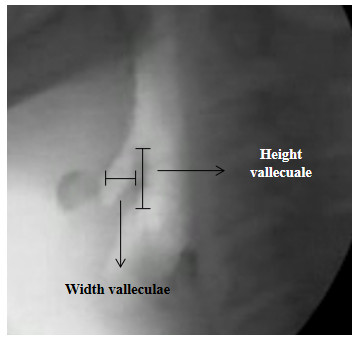
Area of the valleculae.

**Figure 3:**
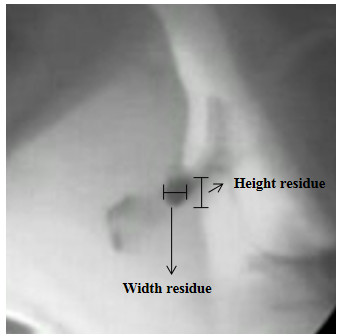
Area of residue.

1. Pharyngeal transit time – PTT [27]: the time interval (in seconds) from the bolus head passing through the ramus of the mandible (event 1) until the bolus tail passes through the cricopharyngeal sphincter (event 2). In order to determine this measure, each one of the two events was identified on the digital images. The conversion to seconds was performed, considering that for the used software each frame corresponds to 0.033 seconds;

2. Duration of the tongue base contact with the posterior pharyngeal wall – TBC [27]: the time interval (in seconds) from the first (event 1) until the last (event 2) contact of the tongue base to the posterior pharyngeal wall during swallowing. In order to determine this measure, each one of the two events was identified on the digital images. The conversion to seconds was performed, considering that for the used software each frame corresponds to 0.033 seconds;

3. Valleculae Residue Ratio – VRR [26]: perceptual two-dimensional ratio based on the analysis of the height × width of the valleculae after the first swallow. In order to determine the VRR the following measurements were performed: (a) area of the valleculae – this measure is obtained by using an area function (height × width). The image of the valleculae prior to swallowing is obtained by freezing the digitalized image on the computer screen. Measurements (height and width), in millimeters, were performed on the actual computer screen using a digital caliper. The vallecuale height was considered the distance from the tip of the epiglottis perpendicular to the base of the valleculae and the width was considered the widest horizontal portion of the valleculae (Figure 2); (b) area of the residue – this measure is obtained by using an area function (height × width) of the residue inside the valleculae. In order to perform this measurement, the image of the valleculae after the first swallowing of each one of the tested volumes and consistencies is frozen on the computer screen. The marker indicating the end of a swallow was the termination of hyoid motion post first swallow [28,29]. Measurements (height and width), in millimeters, were performed on the actual computer screen using a digital caliper. The residue height was considered the distance from the base of the valleculae residue perpendicular to the residue top and the residue width was considered the widest horizontal portion of the valleculae residue (Figure 3); (c) The VRR was determined by expressing the area of the residue in the valleculae relative to the area of the available space in the valleculae as shown below:

In order to guarantee reliability of the performed measurements, all images were analyzed on the same computer (notebook Dell Inspiron 1440) with the zoom tool at 100%. After determining the percentage of the valleculae occupied by the residue, the severity was determined as shown on Table 1:

4. Penetration/Aspiration [30]: penetration/aspiration was determined by using an 8-point multidimensional perceptual scale. Depth of bolus invasion into the airway is a major dimension. Scores were attributed as follows: material does not enter the airway (1); material enters the larynx but stays above the vocal folds and is expelled from the airway (2); material enters the airway, stays above the vocal folds and is not expelled from the airway (3); material enters the airway, touches the vocal folds and is expelled from the airway (4); material enters the airway, touches the vocal folds and is not expelled from the airway (5); material enters the airway, passes below the vocal folds and is expelled from the airway (6); material enters the airway, passes below the vocal folds and is not expelled from the airway; material enters the airway, passes below the vocal folds and no effort is made to expel it. Scores 2 through 8 is assumed to be a more severe sign of dysphagia. Aspiration is scored 6, 7, or 8. Penetration is scored 2, 3, 4, or 5.

**Table 1:** Severity scale for valleculae residue

**Severity**	**Percentage of residue in vallecular space**
Normal	<3
Mild	≥3 a <25
Moderate	≥25 a <55
Severe	≥55

### Measurement of reliability

Inter- and intrarater reliability was measured using weighted kappa, which corrects for the effect of chance and bias. By definition, weighted kappa ranges from 0 (chance agreement) to 1 (complete agreement).

Patient and control subject videofluoroscopic studies were randomly analyzed (20% of the total VFSS samples) by three independent speech and language pathologist who were blinded to subject status (COPD vs control subjects) and were not involved in performing the swallowing study. Kappa was calculated for the residue ratio after the numeric measurements had been categorized as percentages. The interjudge and intrajudge reliability ranged from 0.75 to 0.86 and 0.79 to 0.87 respectively.

### Data analysis

All quantitative data were entered in a SPSS 17.0 database. The Kolmogorov-Smirnov test was used to verify data distribution. The Mann–Whitney test was used for within and between-group comparisons and the Spearman rank correlation coefficients examined any linear association among selected variables. Pearson's chi-squared test was used to compare categorical variables and the Friedman two-way analysis of variance by ranks was used to detect differences across multiple test attempts. The adopted significance level was of 5% for all analysis.

## Results

Table 2 shows the characterization of age, body mass index and spirometry (FEV_1_) of COPD patients and healthy individuals. The age and BMI were similar between patients with COPD and healthy controls, whereas FEV_1_ differed significantly between the two groups. Regarding the spirometric classification of severity, patients presented the following distribution: 2 at stage I (mild); 2 at stage II (moderate); 11 at stage III (severe); and 5 at stage IV (very severe).

**Table 2:** Characterization of participants

**Variables**	**COPD mean(SD)**	**Control mean(SD)**	**p-value**
Age, years	59(4.1)	59(4.4)	0.880
BMI, Kg/m^2^	26.8(7.1)	28.5(5.9)	0.090
FEV_1_	43.3(20.9)	101(11.9)	0.000*

Legend: COPD – chronic obstructive pulmonary disorder; SD – standard deviation; BMI – body mass index; FEV_1_ - forced expiratory volume during the first second; * - significant results.

Participants, i.e. stable COPD patients and healthy controls, did not present any signs of penetration-aspiration for any of the tested consistencies, receiving a score of 1 on the penetration-aspiration scale. Tables 3 and 4 shows between-group comparisons for the PTT, TBC and distribution of individuals among the different severity levels of valleculae residue. Patients with COPD presented longer PTTs during the ingestion of 10 ml of the liquid consistency and during the ingestion of the paste consistency. Regarding the TBC, COPD patients also presented longer durations for the liquid (i.e. 5 ml) and paste consistencies. No significant difference was observed for the distribution of individuals among the different valleculae residue severity levels.

**Table 3:** Between-group comparison for transit times (in seconds)

		**PTT mean(SD)**	**U**	**p-value**	**TBC mean(SD)**	**U**	**p-value**
3 ml Liquid	COPD	1.22(1.26)	135.5	.080	.62(.27)	148.5	.162
	Control	.81(.29)			.55(.30)		
5 ml Liquid	COPD	1.32(.87)	136.0	.083	.95(.79)	94.0	.004*
	Control	1.04(.60)			.51(.24)		
10 ml Liquid	COPD	1.25(.67)	82.5	.001*	.93(.58)	134.0	.070
	Control	.96(.63)			.66(.39)		
Paste	COPD	1.44(1.98)	107.5	.012*	.92(.45)	119.0	.028*
	Control	.80(.25)			.62(.24)		
Solid	COPD	1.56(2.07)	141.0	.110	.56(.11)	153.0	.201
	Control	1.02(1.04)			.52(.12)		

Legend: COPD – chronic obstructive pulmonary disorder; SD – standard deviation; PTT – pharyngeal transit time; TBC – duration of tongue base contact with the posterior pharyngeal wall; * - significant results.

**Table 4:** Between-group comparison for valleculae residue

		**None (n)**	**Mild (n)**	**Moderate (n)**	**Severe (n)**	**p-value**
3 ml liquid	COPD	12	7	1	-	.322
	Control	15	3	2	-	
5 ml liquid	COPD	12	4	3	1	.695
	Control	11	6	3	-	
10 ml liquid	COPD	12	7	-	1	.249
	Control	11	6	3	-	
Paste	COPD	13	6	1	-	.809
	Control	13	5	2	-	
Solid	COPD	16	3	1	-	.766
	Control	16	2	2	-	

Legend: COPD – chronic obstructive pulmonary disorder; n – number of individuals.

Spearman correlation coefficients were used to examine whether baseline characteristics correlated with transit time variables. Significant correlations were observed not only for COPD patients, but also for healthy individuals. Regarding the different food consistencies, the following significant correlations were observed:

• 3 ml of liquid consistency – for healthy individuals the longer the PTT the longer the TBC (Table 5);

• 5 ml of liquid consistency – for healthy individuals the higher the BMI the longer the TBC, and the longer the PTT the longer the TBC; for COPD patients the more severe the disease the shorter the PTT (Table 6);

• paste consistency – for healthy individuals the higher the BMI the longer the TBC (Table 7).

**Table 5:** Spearman correlation coefficient for 3 ml liquid

		**COPD**			**Control**		
		**VEF**_**1**_	**BMI**	**PTT**	**VEF**_**1**_	**BMI**	**PTT**
PTT	r	.201	.760		.045	.022	
	p-value	.395	.751		.850	.928	
TBC	r	.359	.185	.311	.080	.365	.582
	p-value	.120	.429	.183	.736	.113	.007*

Legend: COPD – chronic obstructive pulmonary disorder; FEV_1_ - forced expiratory volume during the first second; BMI – body mass index; PTT – pharyngeal transit time; TBC - duration of tongue base contact with the posterior pharyngeal wall; * - significant results.

**Table 6:** Spearman correlation coefficient for 5 ml liquid

		**COPD**			**Control**		
		**VEF**_**1**_	**BMI**	**PTT**	**VEF**_**1**_	**BMI**	**PTT**
PTT	r	.569	.335		.096	.025	
	p-value	.009*	.149		.686	.916	
TBC	r	.088	.150	.118	.360	.551	.473
	p-value	.712	.529	.620	.120	.012*	.035*

Legend: COPD – chronic obstructive pulmonary disorder; FEV_1_ - forced expiratory volume during the first second; BMI – body mass index; PTT – pharyngeal transit time; TBC - duration of tongue base contact with the posterior pharyngeal wall; * - significant results.

**Table 7:** Spearman correlation coefficient for pasty consistency

		**COPD**			**Control**		
		**VEF**_**1**_	**BMI**	**PTT**	**VEF**_**1**_	**BMI**	**PTT**
PTT	r	.330	.109		-.074	.131	
	p-value	.155	.649		.757	.583	
TBC	r	.154	.121	.386	-.095	.503	.216
	p-value	.517	.612	.093	.691	.024*	.360

Legend: COPD – chronic obstructive pulmonary disorder; FEV_1_ - forced expiratory volume during the first second; BMI – body mass index; PTT – pharyngeal transit time; TBC - duration of tongue base contact with the posterior pharyngeal wall; * - significant results.

Within group comparisons were also performed in order to verify possible differences in pharyngeal transit times and in durations of tongue base contact with the posterior pharyngeal wall among the tested volumes (Table 8). A significant difference was observed only for the group of COPD patients when considering TBC. Multiple comparisons of TBC for COPD patients however, indicated no significant differences among the tested volumes. A trend to significance was observed for the following comparsions: 3 ml versus 10 ml (p = .060) and 10 ml versus solid (p = .069). In both situations, longer durations of TBC were observed during the swallow of 10 ml water (0.62 s for 3 ml water; 0.93 s for 10 ml water; and 0.56 s for solid).

**Table 8:** Within group volume comparisons for PTT and TBC

**Group**	**Variable**	**3 ml**	**5 ml**	**10 ml**	**Paste**	**Solid**	**p-value**
COPD	PTT	1.22(1.26)	1.32(0.87)	1.25(0.67)	1.44(1.98)	1.56(2.07)	.720
	TBC	0.62(0.27)	0.95(0.79)	0.93(0.58)	0.92(0.45)	0.56(0.11)	.004*
Control	PTT	0.81(0.29)	1.04(0.60)	0.96(0.63)	0.80(0.25)	1.02(1.04)	.291
	TBC	0.55(0.30)	0.51(0.24)	0.66(0.39)	0.62(0.24)	0.52(0.12)	.243

Values expressed by mean and standard deviation; Friedman two-way analysis of variance by ranks.

Legend: COPD – chronic obstructive pulmonary disorder; PTT – pharyngeal transit time; TBC - duration of tongue base contact with the posterior pharyngeal wall; * - significant results.

## Discussion

To the best of our knowledge, this is the first clinical study providing detailed analysis of valleculae residue in stable patients with COPD and with no swallowing complaints. The data suggests that the risk for aspiration in COPD population is not limited to the presence of valleculae residue.

Valleculae residue is an important indicator of swallow efficiency and is a significant part of swallow studies [31]. Where large quantities of valleculae residue exist, the individual is at increased risk of aspirating residue during respiration after swallowing [26]. Videofluoroscopic swallow studies have demonstrated that when there is insufficient contact between base tongue and posterior pharyngeal wall, residue remains in the valleculae [26]. In our study, COPD patients presented longer durations of tongue base contact with the posterior pharyngeal wall. Studies by Mokhlesi et al. [11] reported reduced laryngeal elevation during swallow in COPD and an overall altered swallow physiology in the disease. As COPD patients tend to present delayed swallowing reflex and problems with lingual and pharyngeal peristalsis [16,20], we suspect that this mechanism may work as a protective physiologic swallowing maneuver, explaining the absence of swallowing complaints and aspiration in our study.

The same hypothesis could account for the longer duration of the pharyngeal transit times in COPD patients. Because the pharynx is a shared pathway for food and air intake, pulmonary protection necessitates a high level of coordination between respiration and swallowing [13]. Palouski et al. [32] have suggested that longer duration of the pressure waves are related to longer bolus transit times and higher pharyngeal residue. According to the authors, these correlations indicate that longer durations of the pressure waves are associated with worse swallow function, suggesting that a longer duration of pressure is need to initiate and maintain bolus transit in the context of reduced pharyngeal pressure. In our study, the longer pharyngeal transit times could mean, at least in stable patients with COPD, that breathing/swallowing events would have more time to reach coordination before the swallowing reflex itself is triggered. The same would not be true for patients with COPD exacerbations. Since exacerbations of COPD are characterized by acute-on-chronic deterioration in respiration [1], patients are most likely to have difficulties in prolonging swallowing events especially those events that have a close relationship to bringing respiration to a halt.

Another point that we would like to comment on, is that pharyngeal transit times were longer for the larger liquid volumes and for the paste consistency. Consistent with previous reports, textural properties (i.e. viscosity) and volumes of test foods can have an impact on the sensory motor aspects of swallowing and therefore affect some of the assessed durational parameters [33,34]. The literature suggests that by increasing the bolus volume the duration of swallowing events is prolonged (i.e. pharyngeal and laryngeal). It seems that this phenomenon becomes more evident if individuals already present alterations in swallowing [35].

Although not evaluated in the present study, decreased laryngopharyngeal sensitivity must also be taken into account. Recently, studies of Clayton et al. [21] have reported that the impairment of swallowing shown by patients with COPD is possibly caused by a reduction in laryngopharyngeal sensitivity. The authors conclude that patients with COPD have significantly less mechanosensitivity in the laryngopharynx that may be related to the effects of widely used medication on the laryngopharyngeal sensation and also to laryngeal edema caused by chronic coughing. One might argue that these factors may have an influence on the overall sensitivity of the oral tract and therefore could affect the overall swallowing process. Although we strongly believe that physiological adaptations would respond for the longer durations of measured transit times, the hypothesis of decreased oral tract sensitivity should be further investigated.

Our study found no significant difference between stable COPD patients and their healthy controls for valleculae residue. We believe that age influenced our results, in the sense that no difference was observed between the groups as we had expected. Prior studies [36] have described the presence of valleculae residue (pharyngeal retention) in healthy older adults and elderly who are not aware of this pharyngeal pooling [37]. A study by Cook et al. [36] showed that although pharyngeal clearance is nearly complete in young asymptomatic subjects, this is not true in asymptomatic (non-dysphagic) elderly. In the aged, pharyngeal residuals ranged widely from 1 to 13%. Tracy et al. [38] found post-swallow residue to be limited to coating of the tongue base and the valleculae. The mechanism responsible for the development of pharyngeal retention in asymptomatic older individuals are not well known. According to the literature [39-41], different parameters may be involved such as amplitude of pharyngeal contraction, pharyngeal shortening, tongue driving force and hypopharyngeal suction pump. Future studies should include the investigation of swallowing in different age groups of COPD patients.

Our study had some limitations. First, patients with COPD were heterogeneous. Although all patients included in the study were in a stable condition, they presented different spirometric classification of severity. One would expect valleculae residue to have a greater impact and consequence in more severe forms of COPD due to the interaction between lung deterioration and swallowing biodynamic. This point should be included in future research. Secondly, the method used to quantify pharyngeal residue is applied only to the valleculae, with no corresponding measurement for the piriform sinuses. It has been well described that material retained in the piriform sinuses also constitutes a risk for post-swallow aspiration [42]. Moreover, the VRRS is related to the method used for calculating area (linear height by linear width). Recently, Pearson et al. [43] proposed an image-based measurement of post-swallow residue (the Normalized Residue Ratio Scale) based on the analyses of circumscribed area ratios (pixel-based). Although this method still needs further investigation, it is a promising tool to characterize residue in the valleculae and piriform sinuses.

Even though dysphagia is part of the symptoms presented by COPD patients, little emphasis is given to the early identification of swallowing alterations; attention is only given when the patient is already severely compromised. Treatment of COPD involves lifestyle modifications. Early identification of swallowing disorders enables early oral rehabilitation (i.e. compensatory strategies, diet modification), which in turn could decrease the risk of aspiration pneumonia. Finally, future studies are necessary in order to investigation the deterioration process of swallowing in this population and how and which rehabilitation processes can maintain this function healthy for a longer period of time.

## Conclusions

This study adds to the growing body of literature suggesting that stable COPD patients may present physiological adaptations as a protective swallowing maneuver to avoid aspiration/penetration of pharyngeal contents. Moreover, valleculae residue cannot be seen as an isolated factor when trying to explain swallowing alterations in this population.

## Abbreviations

COPD: Chronic obstructive pulmonary disease; FEV1: Forced expiratory volume during the first second; BMI: Body mass index; VFSS: Videofluoroscopic examination; PTT: Pharyngeal transit time; TBC: Duration of tongue base contact with the posterior pharyngeal wall; VRR: Valleculae residue ratio.

## Competing interests

The authors declare no competing interests.

## Authors’ contributions

All authors have made substantial intellectual contributions to the conception and design of the study and the analysis and interpretation of the data. All authors have been involved in drafting the manuscript and revising it critically for important intellectual content. All authors read and approved the final manuscript.

## Pre-publication history

The pre-publication history for this paper can be accessed here:

http://www.biomedcentral.com/1471-2466/14/62/prepub
